# Adult Ileo-Ileo-Caecal Intussusception: Case Report and Literature Review

**DOI:** 10.1155/2012/789378

**Published:** 2012-12-17

**Authors:** Sanjeev Singhal, Anu Singhal, Pankaj K. Arora, Rahul Tugnait, Bishwanath Tiwari, Pawan Malik, Ankur Subhash Dhuria, Vineet Varghese, Mriganka Deuri Bharali, Singh Chandrakant, Pushkar, Vishnu Panwar, Amit Ballani, Neeti Gupta, Vijay Kumar Ramteke

**Affiliations:** ^1^Department of General Surgery, Northern Railway Central Hospital, Basant Lane, New Delhi 110055, India; ^2^Department of Radiology, ESI Model Hospital and PGIMSR, Basaidarapur, Ring Road, New Delhi 110015, India; ^3^Department of Anaesthesiology, Northern Railway Central Hospital, Basant Lane, New Delhi 110055, India; ^4^Department of Radiology, Northern Railway Central Hospital, Basant Lane, New Delhi 110055, India; ^5^Department of Casualty and Obstetrics & Gynaecology, Northern Railway Central Hospital, Basant Lane, New Delhi 110055, India; ^6^Medical Directorate, Indian Railways, Rail Bhawan, Rafi Marg, New Delhi 110001, India

## Abstract

Adult intussusception is a rare entity accounting for only 5% of all intussusceptions and causes approximately 1% of all adult intestinal obstructions. Unlike paediatric intussusceptions which are usually idiopathic, there is usually a lead point pathology which might be malignant in up to 50% cases. We present an unusual case of adult intussusception which was not diagnosed on any investigation including computerized tomographic (CT) scan and magnetic resonance imaging (MRI). It was a case of ileo-ileo-cecal intussusception caused by a large lipoma 38 mm × 43 mm × 61 mm. It was treated by emergency laparotomy for acute intestinal obstruction. A conservative resection with ileostomy was performed with good postoperative recovery.

## 1. Introduction

Intussusception means telescoping of a proximal segment of bowel (intussusceptum) into the lumen of the adjacent distal segment (intussuscipiens). Rarely, a distal segment of the bowel telescopes into the lumen of the adjacent proximal segment, which is known as retrograde intussusception [1]. Intussusception is a relatively common cause of intestinal obstruction in children but a rare and uncommon clinical entity in adults. Adult intussusception (AI) constitutes approximately 5% of all intussusceptions [2], and it accounts for 1–5% of all adult intestinal obstructions [2, 3]. Adult intussusception is usually caused by a tumor acting as the apex of the intussusception. In both small- and large-bowel intussusception, lipoma is the most common benign tumor [4].

## 2. Case Report

The patient was a 55-year-old male, employed as a manual laborer in Indian Railways. Patient presented in emergency with severe abdominal pain, multiple episodes of vomiting, abdominal distension, and obstipation. He had a history of recurrent attacks of colicky abdominal pain, nausea, and vomiting over the last 2 years. There was a history of hospitalization for occasional episodes of severe abdominal pain and distension and obstipation over this period. The frequency of these attacks had been progressively increasing over the last 2 years with the patient requiring weekly admission for the preceding two months before coming to our centre. There was history of loss of weight and appetite. There was no associated history of fever. There was no contributory past or family history. He was a chronic smoker of 80 packet years and a social alcoholic. He was investigated by CT scan of the abdomen and Thorax and MRI of the abdomen during his previous admissions and was diagnosed as a case of hiatus hernia with retroperitoneal fibrosis/diffuse lymphoproliferative disorder. CT chest showed nodularity indicative of infective pathology. However, these investigations were not available with the patient at the time of admission to our hospital. He was normotensive and afebrile but was having tachycardia and tachypnoea (pulse: 108/minute and respiratory rate: 22/minute). His nutritional status appeared adequate. His general physical examination was essentially normal.

His systemic examination of chest, CVS, and CNS revealed no obvious abnormality. On abdominal examination he had a distended abdomen with stretched umbilicus. There were no visible scars. Abdomen was diffusely tender and guarded with no palpable organomegaly. Examination of external genitalia, hernial orifices and renal angles revealed no abnormality. On a digital rectal examination the rectum was empty and ballooned. A provisional diagnosis of acute intestinal obstruction with impending strangulation was made and confirmed on plain X-ray of the chest and abdomen erect and supine which showed grossly dilated small bowel loops and absence of any free intraperitoneal air. His haemogram, biochemical parameters, and urinanalysis done in emergency were within normal limits. 

**Figure 1:**
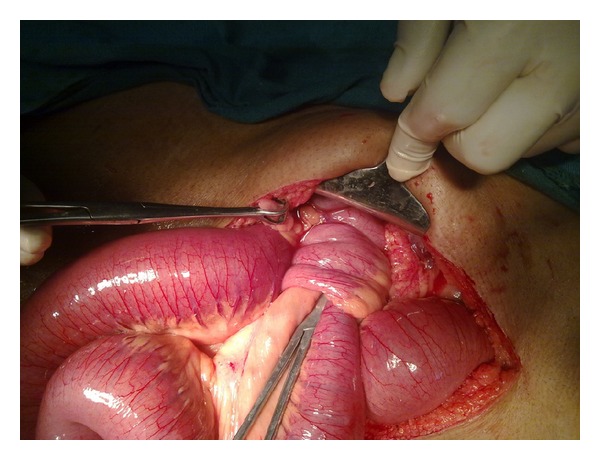
Terminal ileum telescoping into distal ileum and subsequently into ascending colon. Appendix is seen in normal position.

**Figure 2:**
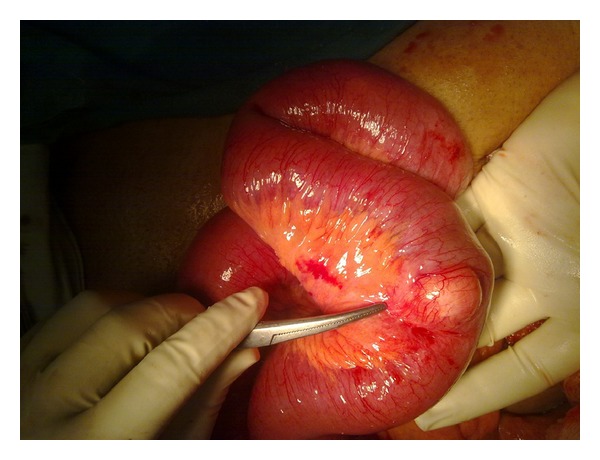
Externally visible tumor which served as a lead point. Note that the bowel is healthy, and mesenetry has no lymph nodes.

**Figure 3:**
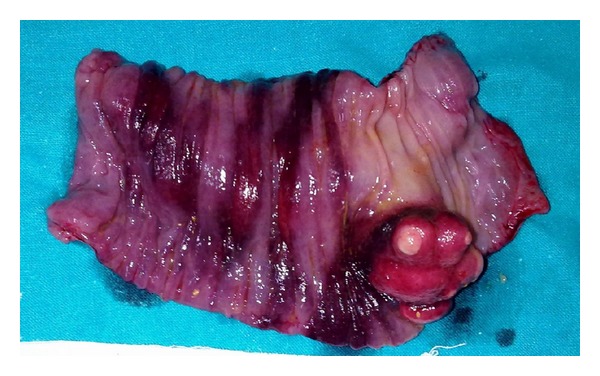
Specimen of resected terminal ileum cut section showing lobulated intraluminal growth.

**Figure 4:**
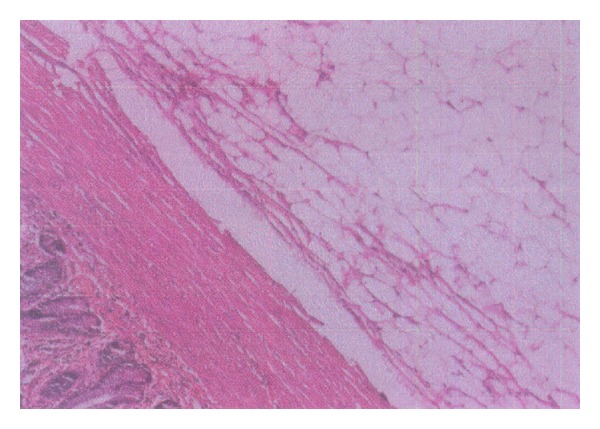
Histopathology showing lipoma.

The patient was taken up for emergency laparotomy without any other investigations as clinically there was an impending strangulation. The abdomen was opened by a midline incision. There was a dilatation of small bowel till the terminal ileum where there was an ileo-ileo-caecal intussusception. The telescoping had started in the terminal ileum and gone through the ileo-caecal valve with the apex extending up to midascending colon (Figure 1). There was no other organomegaly lymphadenopathy or free fluid. The intussusception was reduced by gentle traction and retrograde pressure from the apex. There was a smooth nodule on the lateral wall of the terminal ileum at approximately 40 cm from IC junction (Figure 2). Good bowel viability was ensured. In view of emergency setting, unprepared bowel, lack of preoperative diagnosis, and absence of any lymphadenopathy/nodularity or free fluid it was decided to do a limited resection and bring out an ileostomy and mucous fistula. The ileo-caecal junction and valve were preserved. On resection and gross-examination there was a 38 mm × 43 mm × 61 mm nodule in the wall of ileum (Figure 3) which on histopathology was revealed to be a lipoma (Figure 4). The patient had an uneventful postoperative recovery.

## 3. Discussion

Since its first description in 1674 by Barbette [5] intussusception has been considered to be a disease of infancy and early childhood. Adult intussusception is distinct from pediatric intussusception. It is rare, the condition being found in less than 1 in 1300 abdominal operations and 1 in 100 patients operated for intestinal obstruction. The child to adult ratio is nearly 20 : 1 [6]. In contrast to intussusceptions in children where nearly 80% are idiopathic, a demonstrable etiology is found in nearly 90% of cases in the adult population [7]. This necessitates resection in adults as against reduction in children.

Intussusceptions are classified according to location into: enteric, colonic, and ileocaecal or ileocolic [5]. Enteric and colonic intussusceptions are those that are confined to the small and large intestine, respectively. Ileocolic intussusceptions are defined as those in which ileum prolapses through the ileo-caecal valve into the colon, and these constitute 15% of all intussusceptions. The ileo-caecal valve and the appendix preserve their normal anatomical position, and the organic lesion is usually in the ileum [8]. Our case was one where a part of terminal ileum telescoped into the distal ileum and the whole intussusception then went through the ileo-caecal valve into the caecum and up to the ascending colon.

Small intestinal tumors are rare, accounting for 1-2% of all gastrointestinal tract tumors [9]. Among these, benign tumors are still more rare and account for approximately 30% of all small bowel tumors [10]. The lipomas are rare benign tumors, representing 2.6% of nonmalignant tumors of the intestinal tract [11]. The incidence of intestinal lipomas has been reported between 0.15% and 4.4%. Intestinal lipomas usually occur in older persons, with a slightly increased incidence in females [12, 13]. After gastrointestinal stromal tumors, lipomas constitute the second most common benign-tumor group [10]. Most occur in colon which constitutes from 65% to 75% of cases in comparison with small intestine which constitutes from 20% to 25% [14]. In the small bowel terminal ileum is the commonest site for lipomas [15].

Although they are usually asymptomatic, lipomas larger than 2 cm may cause bowel obstruction, intermittent nonspecific abdominal pain, diarrhea, or bleeding. Furthermore, some lipomas by forming a lead point may cause intussusception, as well [10, 16]. Adult intussusceptions present with nonspecific obstructive symptoms like nausea, vomiting, and abdominal pain. Other symptoms may also be present such as melena, weight loss, fever, constipation, diarrhea, and abdominal mass [17]. In 20% to 50% of cases of adult intussusception, the etiologic agent is a malignancy [18].

Since the clinical picture is vague, varied, and nonspecific, preoperative diagnosis of adult intussusception is rare and essentially radiological. Plain skiagram of the abdomen may reveal features of acute intestinal obstruction [6]. On barium enema colonic lipomas appear as circular, ovoid, well demarcated, and smooth radiolucent masses (because of presence of fat). They show “squeeze sign” due to their fluctuation in size and shape [19].

Ultrasonography is often used to evaluate suspected intussusception as it is cheap, readily available, and noninvasive. The classic features include the “target and doughnut sign” on transverse view and the “pseudokidney sign” in longitudinal view. The major disadvantages are operator dependency and difficulty in image interpretation in presence of air, which is often present in cases of obstruction [6, 20]. The preoperative diagnostic accuracy of ultrasonography is 78.5%. In cases of palpable abdominal mass, the diagnostic accuracy of ultrasonography is even better 86.6% [6]. 

CT scan has been reported to be the most useful imaging technique, with a diagnostic accuracy of 58%–100% and a specificity of 57–71% [6, 20]. On CT, lipomas are seen as homogenous masses, well-circumscribed, ovoid, or round with sharp margins. In addition, they demonstrate characteristic attenuation values between −40 and −120 HU typical of the fatty composition [16]. The CT findings of intussusception are a mass-like lesion, including the inner intussusceptum, an eccentric fat density mass that represents the intussuscepted mesenteric fat, and the outer intussuscipiens, and this appears as a “target” or a “sausage” mass according to imaging plane [4]. CT is excellent in revealing the site, level, and cause of intestinal obstructions and in indicating possible bowel ischaemia. It can give additional information, such as metastasis or lymphadenopathy, which may indicate an underlying pathology [6]. Endoscopy can show a smooth yellow surface with a pedunculated or sessile base or either the “cushion sign” or “naked fat sign” [20].

In view of the uncertain aetiology and diagnosis and high incidence of malignancy (approaching 50%), the treatment of intussusception in adults is invariably surgical resection. However, the extent of bowel resection and the manipulation of the intussuscepted bowel during reduction remain controversial [7, 8, 15]. In contrast to pediatric patients, where intussusception is primary and benign, preoperative reduction with barium or air is not suggested as a definite treatment for adults [7]. The theoretical risks of preliminary manipulation and reduction of an intussuscepted bowel include (1) intraluminal seeding and venous tumor dissemination, (2) perforation and seeding of microorganisms and tumor cells to the peritoneal cavity, and (3) increased risk of anastomotic complications of the manipulated friable and edematous bowel tissue [6–8]. Moreover, reduction should not be attempted if there are signs of inflammation or ischemia of the bowel wall and at age above 60 years [8]. However, several others believe that the risks are theoretical, and gentle reduction should be attempted in selected cases to avoid unnecessary resection of healthy bowel [15]. Endoscopic resection of colonic lipomatous polyps and laparoscopic resection of benign bowel tumors causing ileal and/or ileo-colic intussusception has a role in very selected settings [6, 15].

## 4. Conclusion

Adult intussusception is a rare entity which is distinct from paediatric cases in incidence, aetiology, and management. Ileo-colic intussusception is often caused by lead point pathology which can be a submucous lipoma but may be a malignant lesion thereby necessitating resection and histopathology. Our case had a long history, age of 55 years, and no bowel pathology on CT. Intraoperatively there was viable healthy bowel, and absence of any free fluid, lymph nodes, or nodules in liver. Therefore we attempted reduction by gentle traction and did a limited resection with ileostomy. In adult patients with long history and investigations and intraoperative findings favoring a benign pathology, it is possible to do avoid sacrificing unnecessary length of terminal ileum, more so where it is possible to save the ileo-caecal valve.

## References

[1] Chand M, Bradford L, Nash GF (2008). Intussusception in colorectal cancer. *Clinical Colorectal Cancer*.

[2] Zubaidi A, Al-Saif F, Silverman R (2006). Adult intussusception: a retrospective review. *Diseases of the Colon and Rectum*.

[3] Laws HL, Aldrete JS (1976). Small bowel obstruction: a review of 465 cases. *Southern Medical Journal*.

[4] Mehmet B, Huseyin T, Issam CA, Erkan Y, Ercan K (2012). Ileocecal Intussusception due to a Lipoma in an Adult. *Case Reports in Surgery*.

[5] Se KK, Jae OK, Oh KK (2009). A rare ileal intussusception caused by a Lipoma of the ileum. *Journal of the Korean Surgical Society*.

[6] Rakesh KG, Chandra SA, Rohit Y, Amir B, Panna LS (2010). Intussusceptions in adults: a retrospective interventional series of cases. *Health Renaissance*.

[7] Marinis A, Yiallourou A, Samanides L (2009). Intussusception of the bowel in adults: a review. *World Journal of Gastroenterology*.

[8] Hany B, Samer D (2011). Ileal lipoma—a rare cause of ileocolic intussusception in adults: case report and literature review. *World Journal of Gastrointestinal Surgery*.

[9] Good CA (1963). Tumors of the small intestine. *The American Journal of Roentgenology, Radium Therapy, and Nuclear Medicine*.

[10] Yao T (2001). Primary small intestinal tumors. *Stomach and Intestine*.

[11] Mayo CW, Pagtalunan RJG, Brown DJ (1963). Lipoma of the alimentary tract. *Surgery*.

[12] Ghidirim G, Mishin I, Gutsu E, Gagauz I, Danch A, Russu S (2005). Giant submucosal lipoma of the cecum. Report of a case and review of literature. *Romanian Journal of Gastroenterology*.

[13] Boyce S, Khor YP (June 2009). A colonic submucosal lipoma presenting with recurrent intestinal obstruction attacks. *BMJ Case Report*.

[14] Ashley SW, Wells SA (1988). Tumors of the small intestine. *Seminars in Oncology*.

[15] Akagi I, Miyashita M, Hashimoto M, Makino H, Nomura T, Tajiri T (2008). Adult intussusception caused by an intestinal lipoma: report of a case. *Journal of Nippon Medical School*.

[16] Akyýldýz H, Biri I, Akcan A, Küçük C, Sözüer E (2011). Ileal lipoma: case report. *Erciyes Medical Journal*.

[17] Azar T, Berger DL (1997). Adult intussusception. *Annals of Surgery*.

[18] Briggs DF, Carpathios J, Zollinger RW (1961). Intussusception in adults. *The American Journal of Surgery*.

[19] Shramana M, Vibha K, Kajal KD, Parul G, Nita K (2010). Lipomatous polyp presenting with intestinal intussusception in adults: report of four cases. *Gastroenterology Research*.

[20] Minaya Bravo AM, Vera Mansilla C, Noguerales Fraguas F, Granell Vicent FJ (2012). Ileocolic intussusception due to giant ileal lipoma: review of literature and report of a case. *International Journal of Surgery Case Reports*.

